# A quantitative assessment method for the space design of products based on ergonomics and virtual simulation

**DOI:** 10.1371/journal.pone.0200880

**Published:** 2018-07-23

**Authors:** Ziyue Guo, Dong Zhou, Pengyan Liu, Zhiyi He, Chuan Lv

**Affiliations:** 1 State Key Laboratory of Virtual Reality Technology and System, Beijing, PR China; 2 School of Reliability and Systems Engineering, Beihang University, Beijing, PR China; 3 State Key Defense Science and Technology Laboratory on Reliability and Environmental Engineering, Beijing, PR China; Worcester Polytechnic Institute, UNITED STATES

## Abstract

Work spaces for assembly or maintenance operations are considered in the design stage of products. Maintenance space is a key factor in determining how well a product can be maintained. If the work space is sufficiently considered in the design stage, it will benefit both the user and the maintainer. From a maintenance perspective, this paper proposes a quantitative method to evaluate the maintenance space by virtual simulation and consideration of ergonomics. In this method, maintenance operations are concisely classified into three types. Based on the virtual simulation of the maintenance process and maintainer data, the swept volume of a hand is built using envelope theory. The operation range of the maintainer is derived by establishing a mathematical model for the upper limb. Combining the swept volume and operation range, the maintenance space can be quantitatively evaluated. A concise validation demonstrates that the proposed method is effective for evaluating the maintenance space to improve the space design. The proposed method could be applied to the space design of a complex product.

## Introduction

Maintainability is expressed by the probability that the preventive maintenance (serviceability) or repair (reparability) of an item will be performed within a stated time interval by defined procedures and resources[1]. Accessibility is a key factor of maintainability. It is defined as the ability to approach some areas for maintenance or operation [2]. Accessibility has three aspects: visibility, reachability and maintenance space. Space design is often considered by various industry sectors and users. They can benefit from the good space design of products. Better space design indicates efficient assembly and maintenance processes. Maintenance space is an important factor of a product’s maintainability. Once the design of a product is finished, the features decided by space design become intrinsic properties of a product that reflect how well a product can be repaired when a fault occurs. In a virtual environment, some quantitative evaluation methods exist to measure visibility and reachability, such as the visual cone and envelope ball. However, a proper quantitative method to evaluate the maintenance space is not available [3]. Expert experience and visual estimates are employed to evaluate maintenance space. However, these qualitative methods are with considerable subjectivity [4, 5].

With the development of computer and information technology, virtual simulation has been extensively applied in many fields. As an application of virtual simulation, virtual maintenance (VM) serves an important role in maintainability design, analysis, maintenance training of the product. VM is defined as the application of virtual simulation-based engineering, which enables engineers to evaluate, analyze, and plan the assembly of mechanical systems [6]. VM should satisfy several functional requirements [7, 8]. Lockheed Martin Tactical Aircraft Systems employed VM in the design of the F-16 Fighting Falcon, which was the first time that a physical prototype was eliminated. By building a digital prototype and using a three-dimensional interactive ergonomics and human factors CAD package developed by the University of Pennsylvania (JACK) and Design Evaluation for Personnel, Training and Human Factors (DEPTH), designers analyzed the defects of maintainability and ergonomics to reduce development time and cost significantly. This technology was also employed in F-22 and F-35 fighters [9]. Nanyang Technological University developed a Virtual Maintenance Simulation Desktop System for virtual prototypes, which integrated a new disassembly sequence planning technology and optimization algorithms [10]. CAVE and Workbench have been employed to obtain maintainability-related parameters of a digital prototype [11, 12]. At this stage, many virtual simulation and analysis software programs (such as DELMIA and JACK) have been extensively applied in maintainability design, analysis and verification [9, 13, 14]. In recent years, the technologies of immersive virtual simulation and augmented reality have transformed virtual maintenance into a new field. Using some virtual simulation equipment, researchers and trainers can have more direct interaction with the digital prototype, which is a shift from a straight computer simulation to direct interaction with a digital prototype, especially for maintenance training and maintainability verification [8, 15–20].

The swept volume(SV) is defined as the set of all points touched by a solid (the generator) while performing a motion [21]. In recent years, research on swept volumes primarily focused on the field of solid modeling for automated processing, CNC machining, and the avoidance of moving objects and spatial interference inspection [16]. Martin demonstrated a technique with the necessary complexity to solve the SV and raised basic theoretical issues. To solve the problem, geometric modeling techniques, computer graphics and computer algebra methods should be integrated [22]. Weld described a geometric expression of SV that generates a three-dimensional body[23]. The definition of the point set of a SV is given, which turns the three-dimensional SV problem into a polygonal SV problem. Blackmore et al. [24–26] and Leu [27] provide accurate definitions of the SV. Abdel [28, 29] employed the Jacobian condition to determine the geometric expression of two-parameter or multi-parameter swept volumes and proposed an effective clipping algorithm. Ganter et al. [29] proposed the use of envelope theory to solve the SV problem.

This paper aims to propose an integrated method to make a quantitative assessment for the maintenance space of a product, which considers not only the completeness of a maintenance task but also ergonomics. A model of operation range of upper limbs is built, and the hand operations in a maintenance task are classified into three types. Then, with the data inputs of anthropometry, maintenance process and digital prototype, the maintenance space of a product can be evaluated quantitatively by the proposed method. A method validation proved its effectiveness.

## Method to evaluate maintenance space

### General framework

**Fig 1:**
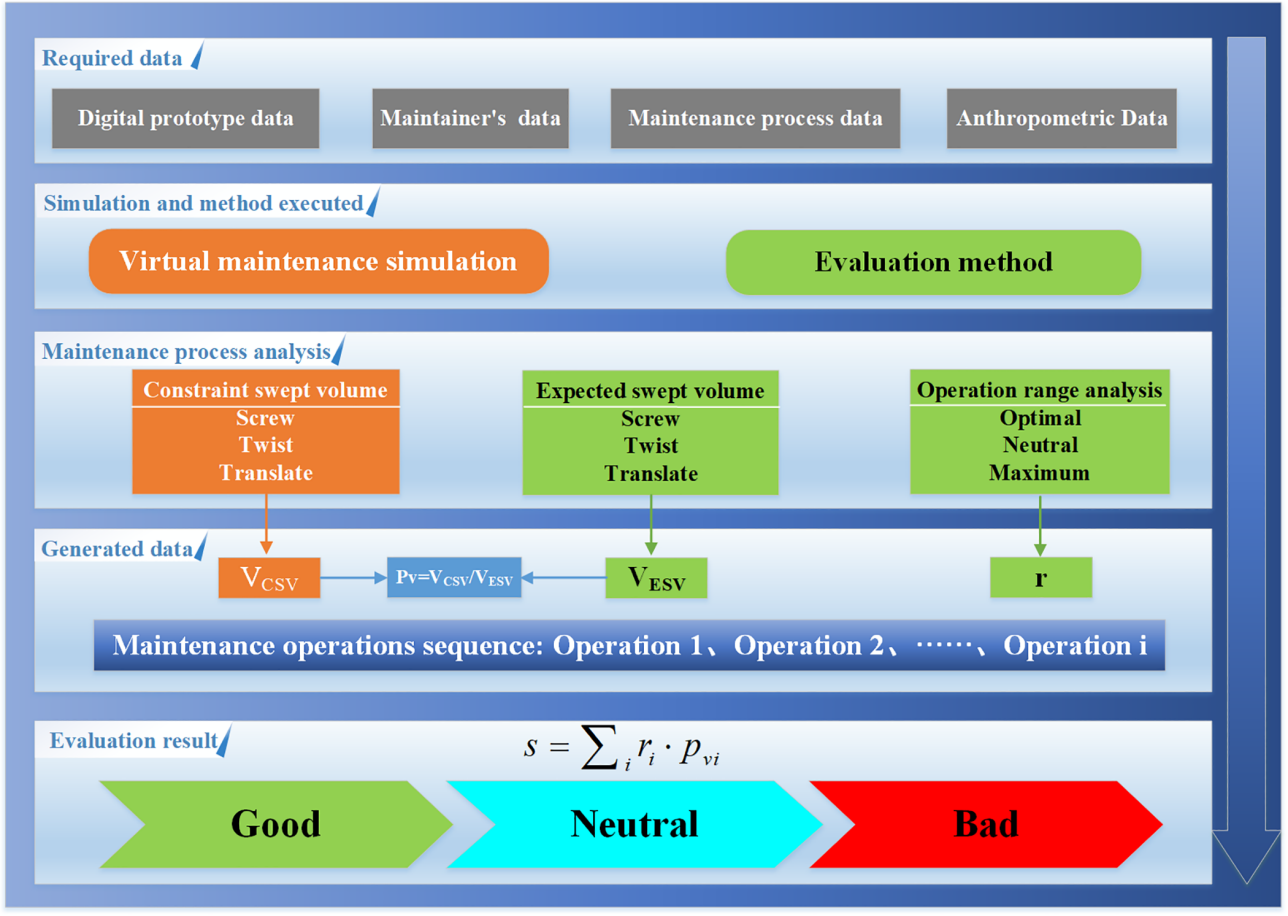
Process of proposed method.

Fig 1 shows the general framework of the proposed method. The proposed method is based on virtual simulation and ergonomics. The required inputs are digital prototype data, anthropometric measurements data of the maintainer, maintenance process data and anthropometric data. The first three data are the basis of virtual simulation. The anthropometric data is part of the input of the proposed method, which will influence the evaluation result. Then, based on our previous study[30], without loss of generality, maintenance operations are divided into three types: screw, twist and translate. According to the anthropometric data and the proposed method, the expected SV(ESV) of these three operations and the operation range of the maintainers can be calculated. The ESV is the ideal SV, which represents the optimal space in which the maintainer can comfortably complete the maintenance task. When the virtual simulation is finished, the constraint SV(CSV) of these three operations could also be obtained. The CSV is the actual SV of the maintainer’s hand, which is the real swept volume constrained by the spatial arrangement of the product.

In the simulation, the value of the CSV (V_csv_) can be obtained. The value of the ESV (V_esv_) and the operation range are obtained by the analysis of maintainer information, ergonomic data and specific operations. By obtaining the operation range and ratio of V_csv_ and V_esv_, the evaluation result can be quantitatively determined. The outputs are the evaluation values, which are classified as the good, neutral or bad. The maintenance analysis and space design analysis can also be performed by the proposed method to guide the improvement of the product.

### Operation range of upper limbs

Maintenance is an activity that requires the involvement of a human. An appropriate maintenance space will enhance the efficiency of the maintenance and the comfort level of the personnel. The main objective of this part is to ascertain the size of an appropriate maintenance operation range.

When the design of products is completed, the maintenance space for a maintainer’s body and operational posture is determined. For the same space, a larger person may feel confined, whereas a smaller person will be comfortable. The maintenance space should consider human factors, such as the operation range of the maintainer. In this paper, the operation ranges that an upper limb can reach consist of the optimal range, the normal range and the maximum range.

In traditional studies, the operation ranges are on a horizontal plane. However, the suitable range for maintenance operations will change with the height of the work surface, the extent of the deviation of the hand from the midline of the body, and the required elevation of the hand. Because machinery and equipment are never entirely located within the same horizontal surface, a three-dimensional space must be considered.

**Fig 2:**
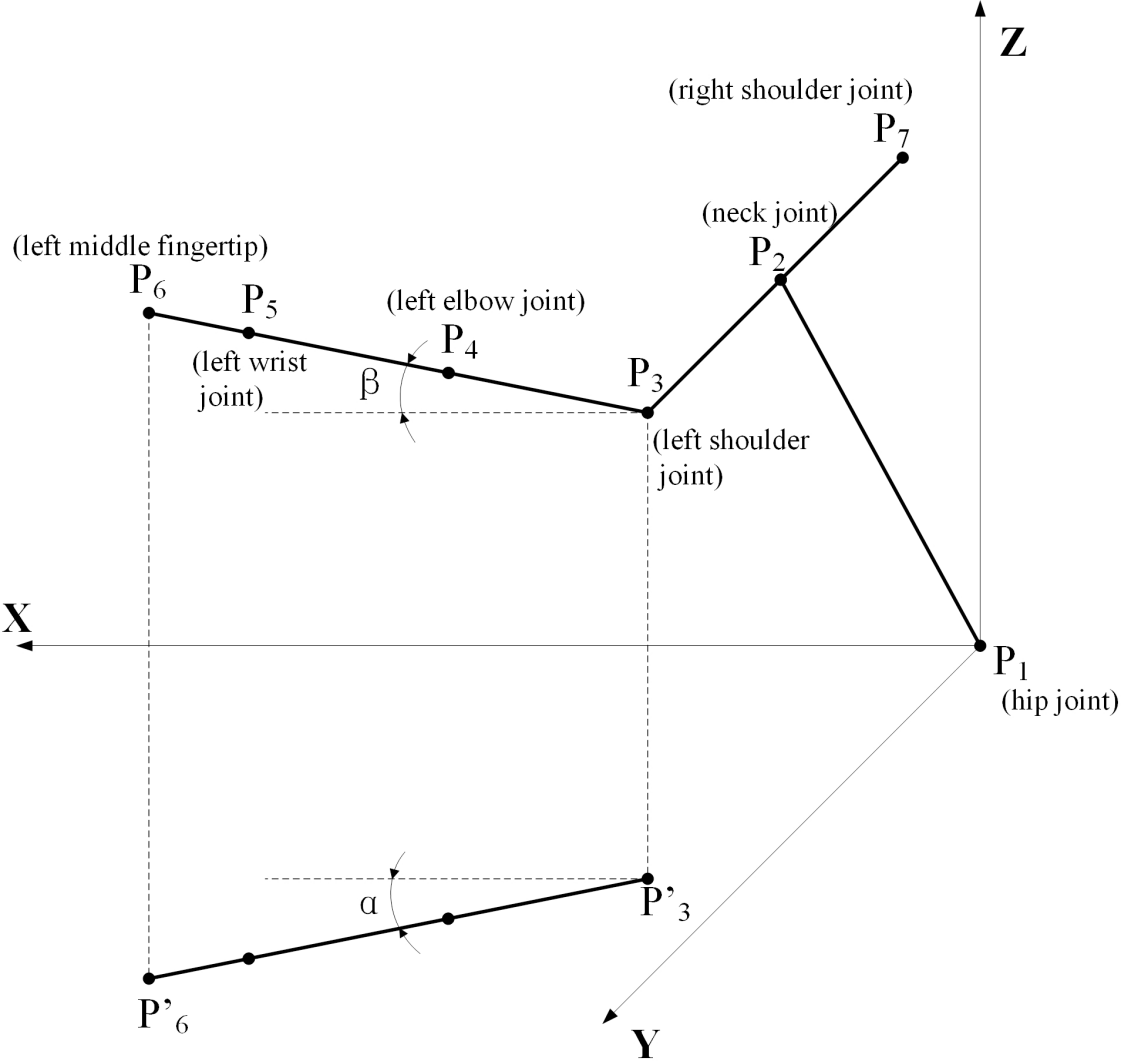
Mathematical model of an upper limb.

The upper limb is the main participant in the maintenance operations. For convenience, we build a model of the upper limb[31], as shown in Fig 2.

We set P_1_ (hip joint) as the origin of the coordinates O (0, 0, 0), and define the front of the body as the X-axis, the left side as the Y-axis, and the vertical direction of the body as the Z-axis. We define P_1_ as the hip joint, P_2_ as the neck joint, P_3_ as the left shoulder joint, P_4_ as the left elbow joint, P_5_ as the left wrist joint and P_6_ as the left middle fingertip. l_ll_ is the length of lower limb, which is not shown in the Fig 2. l_1_ is the length from P_1_ to P_2_. Thus, l_ll_ + l_1_ represents the distance from the vertebra prominens to the floor when the person is in standing position. l_2_ is the length of P_2_ to P_3_, which represents the half-width of the shoulder. l_3_ is the length from P_3_ to P_6_, which represents the length of the upper extremity. α represents the angle between the projections of l_3_ in the XOY plane and the sagittal plane. β represents the angle between the horizontal direction of the left shoulder joint (P_3_) and the left arm when raised or lowered.

Because the left and right hands are symmetrical, the maximum operation ranges on the left side and right side are equivalent. The coordinates of P_6_ are (x,y,z). The body of the maintainer is naturally straight. The operation range can be described as:

$$x=l_{3}\;\cos\;\alpha \;\cos\;\beta$$ (1)

$$y=l_{2}+l_{3}\;\cos\;\beta \;\sin\;\alpha$$ (2)

$$z=l_{1+}l_{3}\;\sin\;\beta +l_{ll}$$ (3)

Because the body is naturally straight, so in equation (3) the l_ll_ should be added to calculate the value of z. The ranges of α and β are

‑4/15π⩽α≤π/2,and

‑π/2≤β≤π/2.

According to ergonomic data[32], when the human arm is straight forward and starts from this position, it can be rotated 48° toward the inside of the body. And the maximum angle of outreach is 90° without considering the space behind the body. The maximum angle of the arm rotating up and down from the horizontal is 90°.

According to data of the human dimensions of Chinese adults, the values of l_1_, l_2_ and l_3_ can be calculated, which are shown in Table 1.

**Table 1:** Values of l_1_, l_2_ and l_3_.

Sex	Male (18–60) ^a^			Female (18–55) ^a^		
Items	P_1_	P_5_	P_50_	P_1_	P_5_	P_50_
l_ll_	892	921	992	826	851	912
l_1_	599	615	657	563	579	617
l_2_	165	172	187.5	152	160	175.2
l_3_	649	675	733	591	614	688

^a^ The data used to calculate l_1_, l_2_ and l_3_ can be found in Chinese national standard GB/T 10000–1988: Human dimensions of Chinese adults.

**Fig 3:**
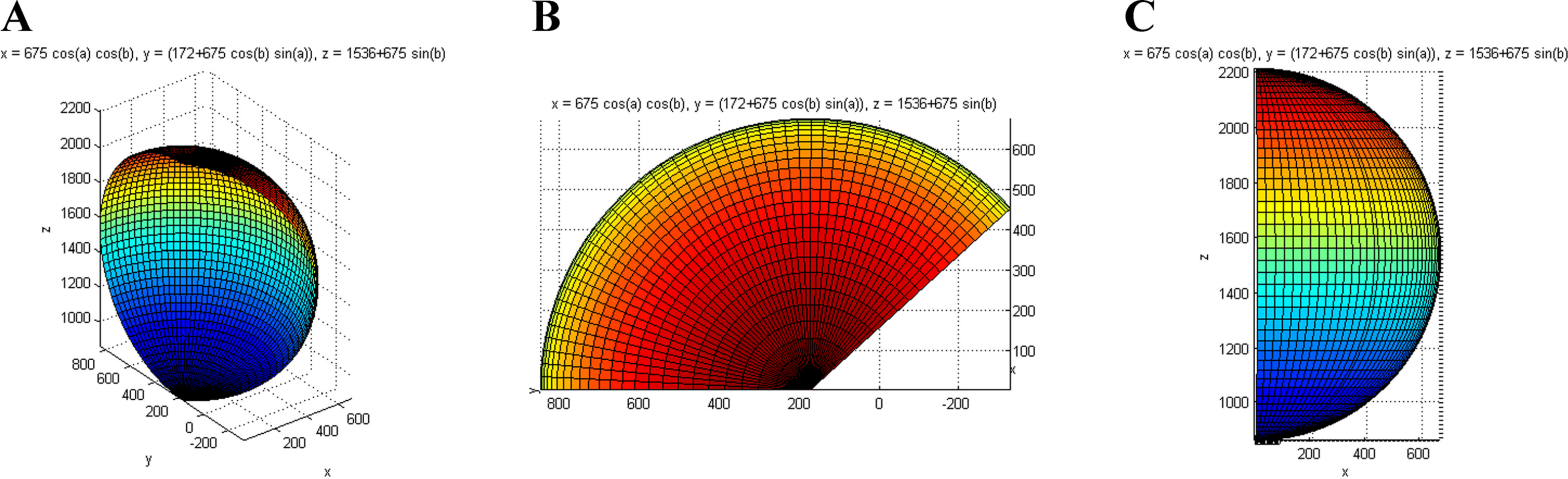
Maximum operation range of the left hand. (A) Isometric view. (B) Top view. (C) Side view.

The design and evaluation of the maintenance space should focus on the higher limit. If the size of space can satisfy the needs of a large-sized maintainer, the needs of a small-sized maintainer are also satisfied. In this paper, males under the 50th percentile are chosen for the maintenance space evaluation. It should be noted that in actual implementations, we should consider the extreme percentile. In this paper, 50th percentile are only used to describe the execution process of the proposed method. Using MATLAB, we can obtain the maximum operation range, which is shown in Fig 3.

With the change of the operating distance, the comfort level of the maintainer and the efficiency of the maintenance will also change. Thus, the optimal, normal and maximum ranges all need to be considered. According to ergonomic data[32], the maximum range is 1.69 times the optimal range, and 1.28 times the normal range. In the optimal range, the maintainer can work for long hours without feeling inconvenienced. Between the optimal range and the normal range, the maintainer can work without feeling tired for a certain period of time. In the range between the normal range and the maximum range, the maintainer will rapidly feel inconvenienced and exhibit poor efficiency.

### Construction of hand

#### Hand size

The size of a maintainer’s hand is a significant factor when designing the maintenance space. Different-sized hands will experience different degrees of difficulty in maintenance operations, which also generate varying quantitative indexes of SVs. Table 2 lists different dimensions of the hands of Chinese males.

**Table 2:** Dimensions of Chinese male hands at different percentiles (mm)^a^.

Item	P_5_	P_50_	P_95_
Hand length	170	183	196
Hand breadth at metacarpals	76	82	89
Index finger length	63	69	76
Finger Ⅱ breadth	15	16	18
Maximum finger Ⅱ breadth	18	19	21

^a^ Data sources: Chinese national standard GB/T 10000–1988: Human dimensions of Chinese adults.

It should be noted that, in some cases, the size of pure hand is only a reference for space design, where the maintainer may wear gloves for some reasons. For example, when replacing the coolant of the aircraft engine, the ground crew should wear gloves to prevent the coolant from harming the human body. The space design around the coolant tube must take into account the space occupied by the gloves, because the maintenances will wear the gloves to unscrew the coolant tube.

#### Different types of hand operations

Once data on the maintainer’s hand are obtained, the CSVs and ESVs of the hand can be determined. According to the differences in the maintenance activities and tools, we divide the operations of the hand into three main types, because a product should be designed to be maintained using standard tools. A bare-handed operation is unusual. These three types can represent common gestures when holding standard maintenance tools. Table 3 describes the three types of operations.

**Table 3:** Three types of operation.

Type	Description
Translate	Parallel movement of hand along a line
Twist	Operation to use a wrench or plier
Screw	Operation to use a screwdriver

The ESV of the hand for each operation presents the optimal space that is required by ergonomic and maintenance needs. The CSVs of the hand can be obtained using the tools in the Human Task Simulation Module of DELMIA to present the maintenance space determined by the actual layout of the product parts [30].

### Evaluation method

**Fig 4:**
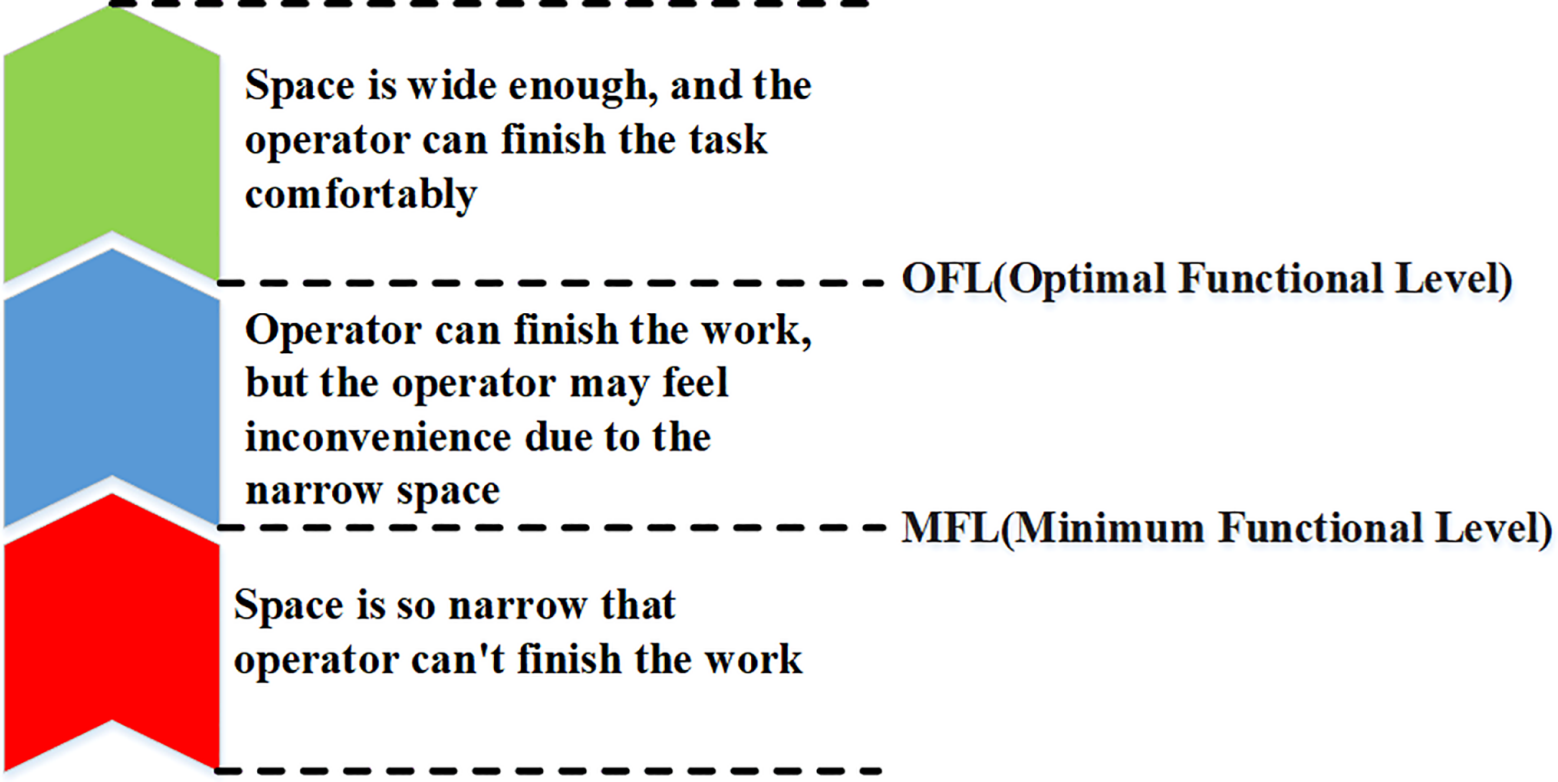
Classification of operating space.

A suitable space for maintenance not only satisfies the most basic needs to complete the maintenance tasks but also makes maintainers feel comfortable. A suitable maintenance space can be defined on two levels: the minimum functional level (MFL) and the optimal functional level (OFL). At the MFL, the space is the minimum-sized space to enable a maintenance task to be performed. However, the maintainer may feel inconvenienced because of the narrow space. At the OFL level, the space is sufficiently broad for the maintainer to accomplish a maintenance task comfortably. The size of the optimal space is dependent on the type of operation, which is shown in Fig 4.

When considering the characteristics of the SV, if a collision occurs in the simulation of a maintenance process, the generated SV cannot represent the actual space occupied by the hand. So, in this study, the presupposition that the motion of the hand is collision-free is made in the simulation of the maintenance process.

The proposed evaluation criteria are developed by considering two aspects: the operation range and the ratio of V_csv_ to V_esv_. In the virtual environment, once the layout of the product, maintenance object and maintainer are confirmed, the CSV can be determined. We define P_v_ as the ratio between the CSV and the ESV,

$$P_{v}=V_{csv}/V_{esv}$$ (4)

where V_csv_ and V_esv_ represent the values of the CSV and the ESV, respectively. For the same operation range, the larger the value of P_v_, the better the maintenance space.

However, the ideal maintenance space is not infinitely large. Bigger is not always better. A reasonable maintenance space satisfies not only the needs of the maintenance task and the comfort of the maintainer but also the requirements of space limitation. Because the size of a product is limited, excessive space allocation for maintenance operations will cause space wastage. For different types of operations, the ESV differs, but is dependent on the body parts that are involved in the operation.

Based on the ergonomic data [32] of the comfortable angles of joints, which are listed in Table 4, the ESV for each operation can be quantified. According to the value of P_v_, the maintenance space evaluation results can be divided into three levels: good, neutral and bad. If the space is less than the MFL, which indicates that the actual space is not able to satisfy the needs for accomplishing the maintenance task, the evaluation results are bad. If the space is between the MFL and the OFL, the maintainer can complete the task, and the evaluation results are neutral. If the space is within the OFL, the maintenance task can be accomplished without compromising the maintainer’s comfort; thus, the evaluation results are good, which indicates that the space design satisfies the ergonomic demands. The evaluation levels are listed in Table 5.

**Table 4:** Maximum angles and comfortable angles of joints.

Joint	Motion modality	Maximum angle	Comfort angle
Neck	Extension/ backward	+40~-35	+12~-25
	Side bending left/right	+55~-55	0
	Twisting left/right	+55~-55	0
Shoulder	Abduction outwards / adduction inwards	+180~-30	0
	Elevation/depression	+180~-45	+15~+35
	Flexion/extension	+140~-45	+40~+90
Wrist	Dorsal extension	30~60	0~30
	Palmar flexion	50~60	0~30
	Radial bending	25~30	0~10
	Ulnar bend	30~40	0~5
Elbow	Bending/stretch	+145~0	+85~+100

**Table 5:** Evaluation criteria for different operations.

Operation type	Evaluation level	P_v_
Screw	Good	>0.67
	Neutral	0.5–0.67
	Bad	<0.5
Twist	Good	>0.5
	Neutral	0.17–0.5
	Bad	<0.17
Translate	Good	>0.74
	Neutral	0.63–0.74
	Bad	<0.63

The previous discussion only considers the relationship between V_csv_ and V_esv_ for one operation; however, the relative positions between the maintenance area and the human body are also important. For the same operation, the comfort level differs with the change in the relative positions between the body and the maintenance area. A complete maintenance task is a combination of a series of operations. Then, the evaluation results of the maintenance space can be calculated as

$$s=\sum_{i}r_{i}∙p_{vi}$$ (5)

where s is the score of the maintenance space evaluation. r_i_ and p_vi_ are the scores of the relative position between maintenance area and body, and the P_v_ of the ith maintenance operation, respectively. According to the relationship among the three operation ranges, if the maintenance area is in the optimal range, the r_i_ is 1.0. If the maintenance area is in the optimal range to the normal range or the normal range to the maximum range, the r_i_ is 0.76 and 0.55, respectively. The evaluation criteria are shown in Table 6.

**Table 6:** Evaluation criteria of maintenance space.

Evaluation level	s
Good	>1.91
Neutral	1.3~1.91
Bad	<1.3, or one or more operation evaluation levels is bad

## A method validation

The purpose of the proposed method is to quantitatively evaluate the maintenance space to assist designers to design operation spaces for maintenance in the digital design stage. Thus, the main issue of method validation is whether the method can accurately and quantitatively reflect the real situation of maintenance space of a product, that is, whether the proposed method can reflect the ergonomic status of a real maintainer and the adequacy of a real maintenance space, to help designers achieve better design for the maintenance space. The method validation will be discussed in this section.

**Fig 5:**
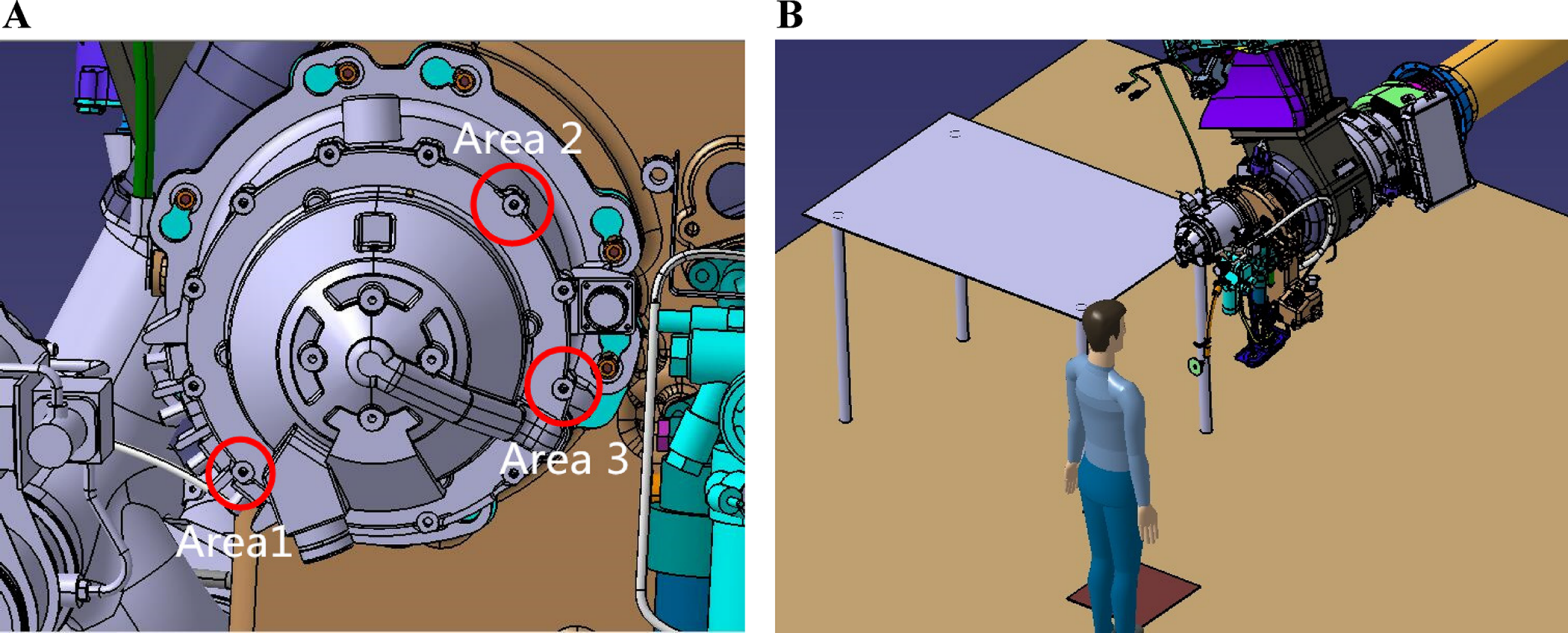
Maintenance areas and scenario. (A) Maintenance areas. (B) General scenario.

Based on the established evaluation method, a maintenance task of an auxiliary power unit (APU) of an aircraft is applied to evaluate the feasibility of the method. The proposed method is utilized to evaluate the maintenance space of an APU quantitatively. The Human Task Simulation Module of DELMIA is selected as the simulation environment. Fig 5 shows the entire maintenance areas and the scenario of the task. The maintenance task is to unscrew the screws which are shown in the red circles. The task of unscrewing the screw is concisely decomposed into three sequential processes: approaching the maintenance target, unscrewing with a wrench, and leaving. These processes correspond to three operations: translate, twist and translate.

**Fig 6:**
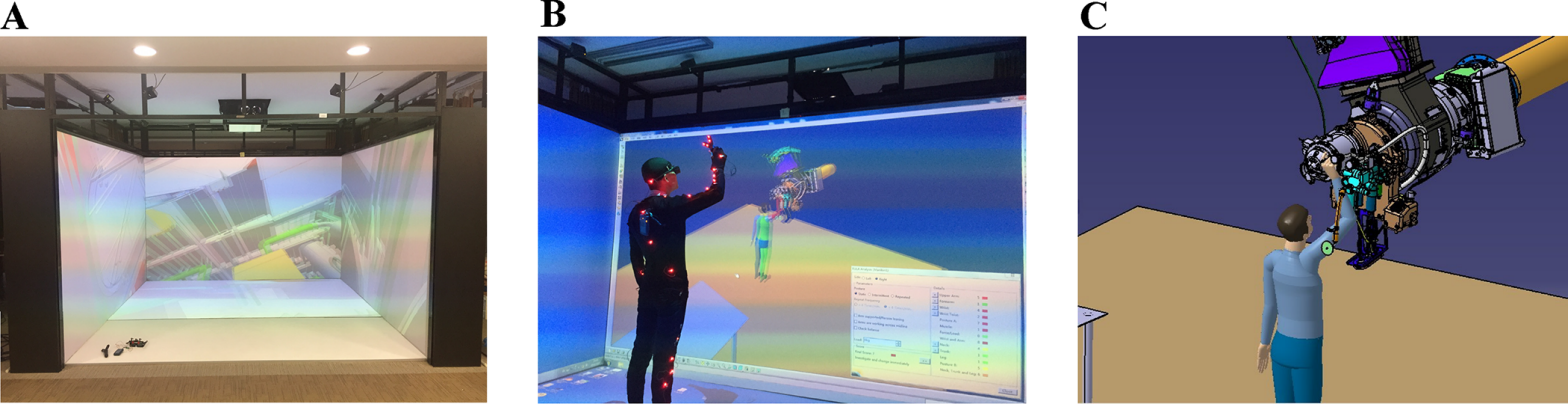
Real task, virtual reality task and simulation task. (A) Virtual reality environment. (B) Virtual maintenance task to obtain data. (C) Simulation of maintenance task.

Fig 6 shows the virtual environment, the virtual task and the simulation task. We employ the virtual task to obtain the required data for real maintenance action. A researcher who wore the motion capture suit performed a real maintenance action; thus, the sensors on the suit can collect posture data. Then based on the rapid motion capture software we developed, the ergonomic status of real human can be analyzed by transmitting the posture data to DELMIA. The Rapid Upper Limb Assessment (RULA) is chosen as the tool to assess the ergonomic situation of operators. It is a useful tool in an ergonomic analysis of the same task [33–36]. The presuppositions are that the surroundings have been simplified without reducing the accuracy of validation, no collision occurs, and the researcher and maintainer in the simulation are P50 persons.

Once the virtual task and simulation are finished, we can obtain the required data, such as ergonomic data and V_csv_ to perform a follow-up analysis.

The evaluation data and results are listed in Table 7 and Table 8, respectively. Detailed discussions of validation will be presented in the next section.

**Table 7:** Evaluation data of the simulation.

Area	Process	Operation	Operation range	V_csv_	V_esv_	P_V_	s	Level
1	Approach	Translate	Normal	1.037	1.037	1.000	2.408	Good
	Unscrew	Twist	Normal	1.342	1.149	1.168		
	Leave	Translate	Normal	1.037	1.037	1.000		
2	Approach	Translate	Maximum	1.037	1.249	0.830	1.384	Neutral
	Unscrew	Twist	Maximum	0.985	1.149	0.857		
	Leave	Translate	Maximum	1.037	1.249	0.830		
3	Approach	Translate	Normal	1.128	1.128	1.000	1.664	Bad
	Unscrew	Twist	Normal	0.217	1.149	0.189		
	Leave	Translate	Normal	1.128	1.128	1.000		

**Table 8:** Evaluation data of real maintainer’s action.

Area	Process	Operation	Operation range	V_csv_	V_esv_	P_V_	s	Level
1	Approach	Translate	Normal	1.037	1.037	1.000	2.497	Good
	Unscrew	Twist	Normal	1.476	1.149	1.285		
	Leave	Translate	Normal	1.037	1.037	1.000		
2	Approach	Translate	Maximum	1.125	1.249	0.901	1.471	Neutral
	Unscrew	Twist	Maximum	1.003	1.149	0.873		
	Leave	Translate	Maximum	1.125	1.249	0.901		
3	Approach	Translate	Normal	1.128	1.128	1.000	1.667	Bad
	Unscrew	Twist	Normal	0.223	1.149	0.194		
	Leave	Translate	Normal	1.128	1.128	1.000		

## Discussion

### Validation of proposed method

**Fig 7:**
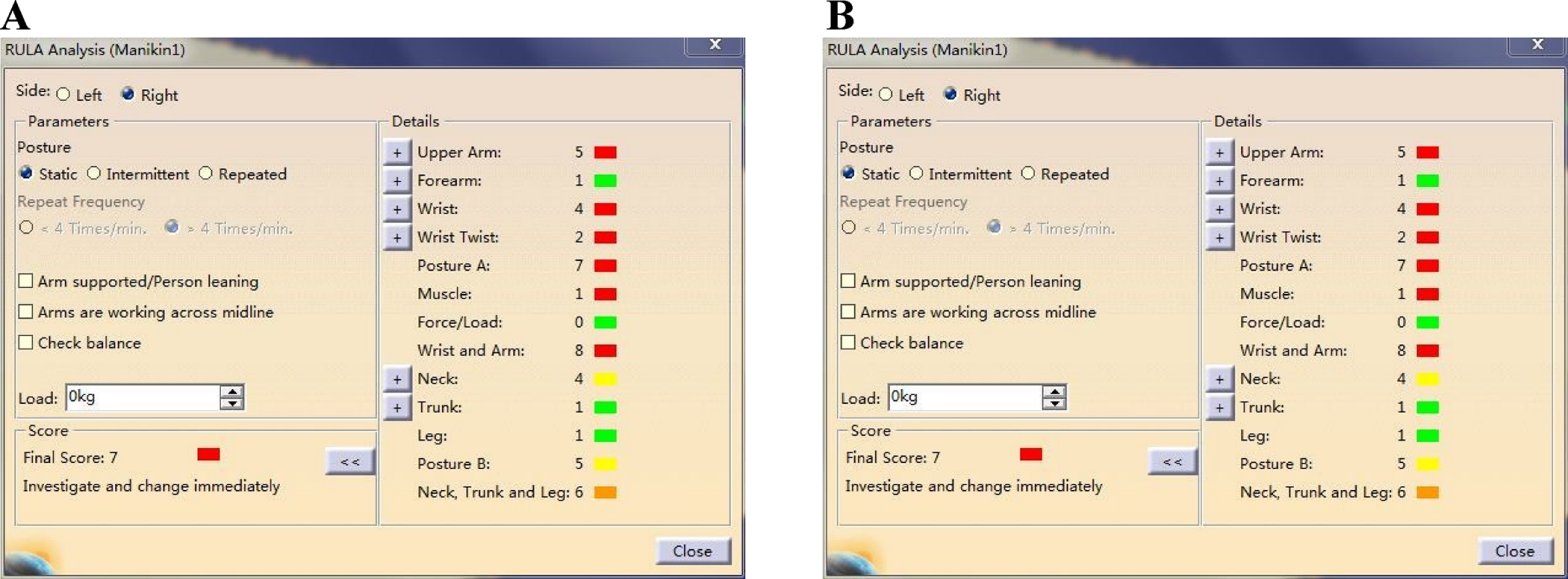
RULA analysis for operators in real task and simulation task. (A) RULA analysis of real maintainer. (B) RULA analysis of simulation task.

Based on the evaluation data of the simulation task of the proposed method and the real operator’s action, which are shown in Table 7 and Table 8, the values of V_csv_, s and P_v_ are similar. The values in Table 8 are slightly higher than the values in Table 7 because the motion capture suit that the researcher wore in the process of gathering ergonomic data occupied some space, which causes the expansion of V_csv_. The errors in s and P_v_ are also within the allowable range. As shown in Fig 7, the RULA analysis that reflects the ergonomic status of an operator indicates that the ergonomic evaluation in a real task and proposed method is also similar. The data analysis has demonstrated that the proposed method has sufficient accuracy and pragmatic values for the real space design of the product. We also invited eight maintainers and eight designers to assess the proposed method and complete questionnaires. Table 9 shows the questionnaire. The first eight respondents were ground crews from airlines or air bases. The last eight responders were designers from aircraft manufacturers. Options A, B, C and D respectively represent that the attitudes of responders towards correlative questions are very satisfied, satisfied, neutral and unsatisfied.

**Table 9:** Questionnaire statistics from eight operators and eight designers.

Personnel No.	Question 1				Question 2				Question 3				Question 4			
	A	B	C	D	A	B	C	D	A	B	C	D	A	B	C	D
1	√					√				√				√		
2		√				√			√				√			
3		√				√			√					√		
4	√				√					√				√		
5	√				√				√				√			
6	√				√				√					√		
7		√					√				√				√	
8	√				√						√		√			
9		√			√				√				√			
10		√				√			√				√			
11		√				√			√					√		
12	√				√				√				√			
13	√				√					√			√			
14	√				√				√				√			
15		√			√				√					√		
16		√			√					√			√			
Question 1: Can the method represent a real ergonomic feeling in actual maintenance operations? Question 2: Can the evaluation results of the proposed method reflect the actual status of the maintenance space or determine if the difference is acceptable? Question 3: Does the proposed method translate to better ergonomics for the operator? Question 4: Is the proposed method useful for the space design of a product?																

The results also demonstrate that the proposed method translates to better ergonomics for the operators and designers.

The following subsections will discuss the maintenance space of each area based on the proposed method.

### Area 1

Area 1 is relatively capacious. The P_v_ values of the translate and twist operations are equal to or greater than 1, which indicates that sufficient space exists for a maintainer to operate. In addition, area 1 is close to the maintainer. All operations are taken in the normal range due to adequate space to operate. The translate operation is a movement in a straight line. For convenience, if V_csv_ is greater than V_esv_, the value of P_v_ is 1. For the twist operation in area 1, few obstacles exist. Thus, the space for the twisting satisfies the OFL. According to the evaluation criteria, s is 2.408, which indicates that the evaluation level of area 1 is good.

### Area 2

The values of P_v_ and s in area 2 are within the neutral level, and the evaluation results are neutral. Although the P_v_ of each operation is good, the operations are within the maximum range. Thus, the evaluation results are neutral. The evaluation results are influenced not only by the size of the actual maintenance space but also by the operation range. The maintainer should stand at a higher position to facilitate the operation, which will yield better evaluation results.

### Area 3

**Fig 8:**
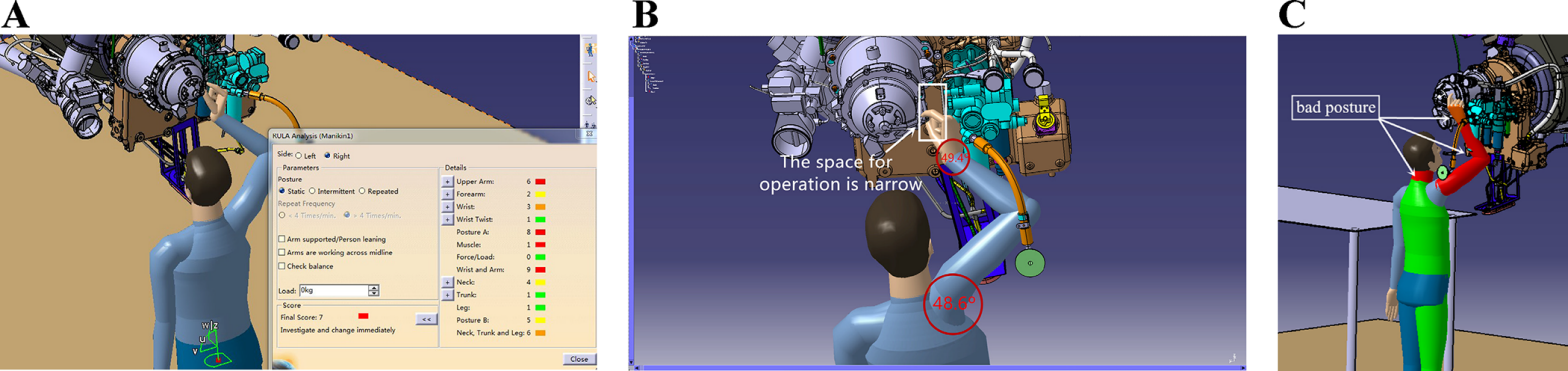
RULA analysis and operation space of Area 2. (A) RULA analysis. (B) Space condition for operating. (C) Active RULA display.

Area 3 is located in a narrow area near the starter and pipelines. The available space for all operations is in the marked rectangular area, which is shown in Fig 8 (B). For the twist operation, the P_V_ is bad, which indicates that the space is too small to complete the unscrewing operation. According to the stated criteria, the evaluation results are bad, even if s is 1.664, which is greater than 1.3. Besides, when performing the maintenance operation in area 3, the maintainer’s wrist joint and shoulder joint are in a bad posture. The maintainer will feel inconvenienced if the posture is maintained for a long time. The dorsal extension angle of the wrist is 49.4°, and the elevation angle of the shoulder is 48.6°, which are outside the comfort angle range of joints. The RULA analysis also demonstrates this conclusion, as shown in Fig 8.

Note that the choice of tools will influence the evaluation result. In this simulation, a ratchet wrench is used to unscrew a screw to test how different tools influence the maintenance space, which requires more space on the vertical plane than a screwdriver. Area 3 has a considerable amount of space in the horizontal plane; therefore, if the tool is a screwdriver, the evaluation results will improve.

### Comparison with the previous study

In our previous study [30], the same auxiliary power unit (APU) of the aircraft is also chosen as the maintenance object to make a case study. But some of the evaluation results differed, as shown in Table 10.

**Table 10:** Evaluation results of the proposed and previous method.

Area 2	Process	Operation	V_csv_	V_esv_	P_V_	s	Level
Proposed method	Approach	Translate	1.037	1.249	0.830	1.384	Neutral
	Unscrew	Twist	0.985	1.149	0.857		
	Leave	Translate	1.037	1.249	0.830		
Previous method	Approach	Translate	1.555	1.555	1.000	0.890	Good
	Unscrew	Twist	1.065	1.512	0.704		
	Leave	Translate	1.555	1.555	1.000		

We can find that the evaluation results have obvious differences between the proposed and previous methods for area 2, because the proposed method considers the motion range of the upper limb. Maintenance is an activity in which the person and machine interact. Therefore, ergonomics should be considered. With the premise that P_v_ is the same, the comfort level of the maintainer is dependent on the variation in the operation range, which explains why the results vary for the same maintenance area. For area 3, poor evaluation results are obtained using both methods due to the narrow space for the twist operation.

By analyzing the process of the disassembly of an APU of the aircraft, we demonstrate how the proposed method is employed to evaluate the maintenance space, determine the limitations of the layout design and provide recommendations for better maintenance. The method proposed in this paper is useful and efficient.

From the case of method validation, we can find some other valuable points to discuss. First point is that the maintenance space in this paper only focus on the space that is occupied by the hand, which is the main body part involving maintenance operation. So the space design for hand should be concentrated at the higher limit. But if the maintenance task is an under-up task, just like the case in A method validation, the space and layout designs should also give consideration to the lower-limit. In that case, the space and the layout of the product should satisfy the reach of a small-sized operator. For example, Swedish Air Force fighter JAS 39 Griffin has good maintainability design, which enables the maintainer to conduct the most maintenance tasks as long as the maintainer stands on the ground.

The second point is that the proposed method is based on the virtual simulation. There are both advantages and disadvantages. With the help virtual simulation on desktop software, we can obtain the maintenance operations quickly. The effectiveness of the space design will improve. But if the producer (usually is the designer) of virtual simulation lacks experience, the simulation may can’t close to the real maintenance process. A certain amount of the differences between virtual simulation and real maintenance process could bring considerable errors to the evaluation results. That is why we let a real person to wear the motion capture suit in the case study. So that the motion data generated by the suit could be used to help the designer eliminate the deviation of simulated manikin.

## Conclusions

In this paper, a method for evaluating the maintenance space in the virtual environment is proposed. Based on the comprehensive consideration of the layout of the product, maintainer and the tools, the proposed method could perform a quantitative evaluation of the maintenance space and provide suggestions. The advantages of the proposed method are as follows: (1) A quantitative method to evaluate the maintenance space is proposed, which provides a credible digital approach which can supplant subjective knowledge. (2) The space design could be conducted in a rapid way in the early design stage of the product. The defect of space design could be found in time, which will save cost by using the digital prototype and virtual simulation.

Compared with the previous study, the proposed method doesn’t consider the operation time. Because the operation time for the same operation is always similar to a well-trained operator [37]. It does not have a strong internal relation between operation time and operation space.

The complexity of the space design remains an ambiguous issue in design departments of the industrial sector. Experience-based design is the common practice, and it is a feasible method for civilian products. But for large industrial equipment or weapons, accurate space design indicates fewer accidents and a higher maintaining efficiency. This paper is based on the corporation with the Chinese aviation and aerospace industry sector. The proposed method has been applied into the maintainability verification of some products. The engineering application has indicated that the quantitative assessment method is capable of helping designers make reasonable and accurate space optimization for products.
